# Fortuitous discovery of ganglionic tuberculosis after conservative treatment of breast cancer: a case report and review of the literature

**DOI:** 10.1186/s12905-019-0780-8

**Published:** 2019-06-18

**Authors:** Aziz Slaoui, Nivine Cherkaoui, Othmane El Harmouchi, Hafid Hachi

**Affiliations:** 1 Department of Gynaecology and Obstetrics, Maternity Souissi, University Hospital Center IBN SINA, University Mohammed V, Rabat, Morocco; 2 Gynaecology Department, National Oncology Hospital, University Mohammed V, University Hospital Center IBN SINA, Rabat, Morocco

**Keywords:** Ganglionic tuberculosis, Breast cancer, Chemotherapy and tuberculosis

## Abstract

**Background:**

Ganglionic tuberculosis is the most common extrapulmonary localization in Morocco. It is still a diagnostic and therapeutic problem especially when the infection is concomitant to the management of a cancer.

**Case presentation:**

Here, we report the uncommon case of a fortuitous discovery of ganglionic tuberculosis in the anatomopathological analysis of an axillary node dissection after conservative treatment of breast cancer for a 29-year-old patient without medical history. Her file was discussed in a multidisciplinary consultation meeting during which several decisions were made. We decided to start her antituberculosis treatment then after three weeks her adjuvant chemotherapy with radiotherapy and hormone therapy. Furthermore, giver her young age, she had an oncogenetic consultation. Despite difficulties of therapeutic compliance, the patient completed her cancer treatments after two years, she also cured of her tuberculosis. Being in remission, she is still on hormone therapy and consults every 3-months as part of her follow-up.

**Conclusions:**

Cancer and tuberculosis presenting simultaneously creates clinical and histopathological difficulties for differential diagnosis and for therapeutic decisions. Anticancer chemotherapy is not an obstacle in anti-tuberculosis treatment but the compliance of patients to receive both at the same time with the associated side effects is one to consider.

## Background

According to the World Health Organization, tuberculosis is a global public health problem and about one-third of the world’s population is infected [1]. In Morocco, 26,000 to 27,000 new cases of all forms of tuberculosis are detected annually and extra pulmonary tuberculosis accounts for 46% of tuberculosis cases, dominated by lymph node tuberculosis [2]. In rare cases, tuberculosis and cancer can be detected at the same time, creating a real therapeutic challenge. Not only in Morocco, Africa, but all over the world and especially in Eastern Europe and the greater part of Asia, surgical and medical oncologists as well as pathologists should be aware of the possibility and associated treatment sequence.

## Case presentation

We hereby present the rare case of a 29-year-old white woman married for 5 years gravida 1 para 1 without significant personal or family history who found during breast self-examination a left breast mass. Then she went to a high medical center where she received breast ultrasound and a mammogram that revealed the presence of a left breast cancer classified 5 in the Breast Imaging Reporting And Data System of the American College Of Radiology, that is to say highly suggestive of malignancy (more than 95%). The radiological report noticed a mammary nodule at the level of the supero-external quadrant of the left breast of 2 cm long axis with the presence of two homolateral axillary lymphadenopathies of 1.2 cm and 0.8 cm. Then she was referred to us and admitted to the National Institute of Oncology in Rabat. The clinical examination confirmed the presence of a mobile mammary mass at the level of the supero-external quadrant of the left breast of 2 cm long, without inflammatory or cutaneous signs, nor mammalian flow, with just one mobile axillary homolateral suspicious ganglion of 1 cm.

First, we performed a micro biopsy with pistol to confirm histologically the presence of the cancer which turned out to be a non-specific infiltrating carcinoma grade 3 (differentiation 3, anisonucleosis 3 and mitotic index 3) of the Elston-Ellis modified Scarff-Bloom and Richardson staging with no intraductal component nor intravascular tumor emboli. She then had a thoraco-abdominopelvic computed tomography as part of her extension assessment, which was negative. Taking into consideration all these elements, we were able to classify the tumor cT1N1M0. We therefore decided to offer conservative treatment to the patient as soon as possible given the diagnosis of cancer at a relatively early stage, which she accepted.

Three weeks later, the patient underwent lumpectomy with ipsilateral axillary dissection. The one-month follow-up showed that the wound had healed well. There was no significant complication in the short term. The histopathology report confirmed the presence of infiltrating ductal carcinoma (WHO 2003) measuring 2.2 cm long axis, grade 3 (differentiation 3, anisonucleosis 2 and mitotic index 2) of the Elston-Ellis modified Scarff-Bloom and Richardson staging, with no intraductal component nor intravascular tumor emboli, with healthy exeresis limits: non-tumorous deep plane at 0.8 cm, healthy lateral margins closest to 0.2 cm; and at the level of axillary dissection 12 N(−)/12 N. The pathological staging of the tumor was therefore pT2N0M0. On the other hand, the histological examination of the axillary dissection revealed the presence of several epitheloid and gigantocellular granulomas centred by a caseous necrosis, typical of follicular ganglionic tuberculosis. The immunohistochemical profile of the tumor could be elaborated and revealed a strong expression of hormone receptors (oestrogen and progesterone), a 30% ki67 without overexpression of HER 2. According to the genomic classification of breast cancer, we could therefore classify this tumor as a luminal tumor A [3].

Her file was discussed in a multidisciplinary consultation meeting during which several decisions were made. First, TB case was notified and Directly Observed Treatment Short course was given.

She received her anti-tuberculosis treatment with a course of 6 months according to the latest WHO recommendations of 2HRZE/4HR (2 months of the Isoniazid, Rifampin, Pyrazinamide and Ethambutol association followed by 4 months of Isoniazid and ethambutol biotherapy) [4]. Three weeks after the beginning of her anti-tuberculosis treatment, she began her adjuvant chemotherapy to prevent locoregional or general recurrence. This treatment was based on 3 courses of four-weekly FEC 100 (5-fluorouracil, Epirubicin, Cyclophosphamide) followed by 3 courses of four-weekly docetaxel. At the end of the chemotherapy she had an external radiotherapy by irradiation of the breast remaining after lumpectomy of 50 Gy with a complement of irradiation on the tumor bed of 15 Gy. Moreover, she also received an anti-estrogen hormone therapy (tamoxifen). Trastuzumab has not been proposed since the tumor does not overexpress HER 2 (human epitheloid growth factor receptor). Finally, given her young age, she had an onco-genetic consultation with search of genes BRCA-1 and BRCA-2, negative income for both.

Despite difficulties of therapeutic compliance, after 2 years the patient completed her cancer treatment and she cured of her tuberculosis. Being in remission, she is still on hormone therapy and consults every 3-months as part of her follow-up.

## Discussion and conclusions

### Breast cancer

According to the American Cancer Society, breast cancer makes up 25% of all cancer diagnoses in women globally [5]. Walters et al. showed in his study that survival for breast cancer is strongly related to the stage of the disease at diagnosis [6]. Unfortunately a recent study found that in Morocco only 32,2% of breast cancer are diagnosed at the local level and the average tumor size at the time of diagnosis is 5 cm [7].

Furthermore, several recent studies have compared conservative with radical treatment and they have demonstrated comparable survival rates and recurrences, while conservative treatment would provide a better quality of life [8–10].

### Ganglionic tuberculosis

Tuberculosis is also a global public health problem and more than 9 million new cases of tuberculosis occur each year [1]. The diagnosis of isolated ganglionic tuberculosis in our patient was made thanks to the histoanatomopathological analysis of ganglion dissection and to the unsuccessful search for associated pulmonary tuberculosis.

Calarasu et al. [11] reported a similar case of a middle-aged woman previously diagnosed with skin cancer, who underwent chemotherapy and later presented with axillary adenopathy of unknown origin, supposedly metastatic, which pathology revealed to be extrapulmonary tuberculosis. Calarasu et al. [11] concluded that immunodepression induced by chemotherapy might lead to reactivation of viable *Mycobacterium tuberculosis* within the macrophage of granulomas. However, in our case the fortuitous discovery of tuberculosis was earlier than any treatment and almost concomitant with the discovery of cancer itself. Its occurrence is fortunately independent, since chemotherapy could have boosted the tuberculosis spread.

### Chemotherapy and tuberculosis

Safdar et al. [12] describe infection like a major cause of morbidity and mortality in patients with cancer. Seo GH et al. [13] studied specifically the tuberculosis incidence rate in patients with malignant tumors compared to the general population and concluded that it is higher even 24 months after the diagnosis and breast cancer is one of the cancers that increased the incidence of tuberculosis the most. A review of literature allowed us to find other authors such as Kamboj et al. [14] and De La Rosa et al. [15] who came to the same conclusion: tuberculosis is more common in cancer patients. Data from the literature suggests that this increase is due to a decrease in host immunity; as a matter of fact, cancer patients may suffer from reactivation of their latent tuberculosis during their treatment because of immunosuppression induced by the disease or by the treatment [16, 17]. Therefore, we recommend that practitioners consider tuberculosis as comorbidity in patients with malignant tumors.

In terms of treatment, most authors recommend starting TB treatment before initiating chemotherapy [18, 19]. Vento et al. [18] recommend starting antituberculosis isoniazid prophylaxis when tuberculin skin test is positive (≥5 mm induration) in a cancer patient. As for Baslmain et al. [19], they warn practitioners of the adverse effects of non-necessary antituberculous treatment but also recommend starting it before chemotherapy.

The specificity of our case turns out to be the chronology of the diagnoses. Indeed, the management of the breast cancer led to the fortuitous discovery of ipsilateral ganglionic tuberculosis. The cause-and-effect relationship that has been revealed in the few studies that have examined the association between both cannot be established in our case. In fact, there is no immunodepression induced by chemotherapy or cancer itself that would explain a reactivation of latent tuberculosis. Furthermore, this turn of events allowed us to take the necessary steps, namely to start antituberculous treatment 3 weeks before starting the adjuvant chemotherapy as recommended in the literature [19].

Cancer and tuberculosis presenting simultaneously creates clinical and histopathological difficulties for differential diagnosis and for therapeutic decisions. The authors suggest also that there is a high index of suspicion for ganglionic TB during investigation of lymph nodes in breast cancer patients, more so in LMICs/developing countries which have a high burden of TB. Anticancer chemotherapy is not an obstacle in anti-tuberculosis treatment but the compliance of patients to receive both at the same time with the associated side effects is one to consider. Education and rigorous follow-up of patients and family are necessary for a successful outcome.

## Data Availability

The datasets used and/or analyzed during the current study are available from the corresponding author on reasonable request.

## Abbreviations

DOTS: Directly Observed Treatment Short course
TB: Tuberculosis
WHO: World Health Organisation
